# Supplementary material for: Widespread selection for extremely high and low levels of secondary structure in coding sequences across all domains of life
Source: Open Biol. 2019 May 29;9(5):190020. doi: 10.1098/rsob.190020 (PMC6544989; doi:10.1098/rsob.190020)
Supplement: Supplementary Figures [file rsob190020supp1.docx]

**Supplementary Figures from Gebert et al. 2019 (doi: 10.1098/rsob.190020).**


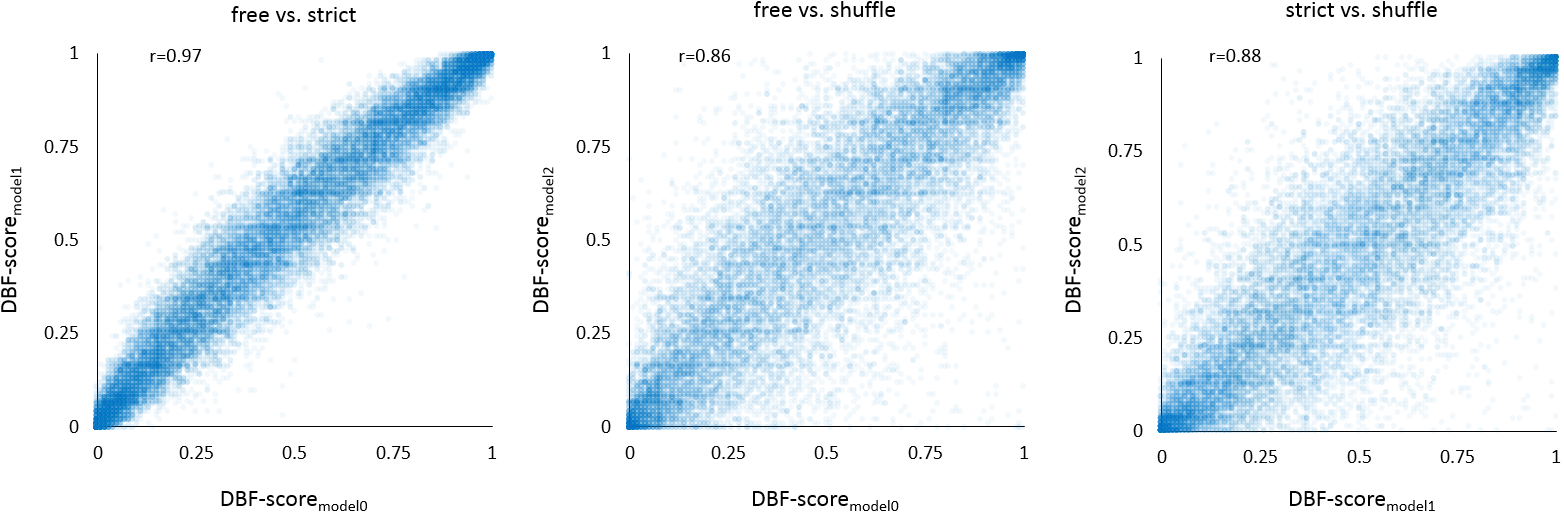


**Supplementary Figure 1.** Pairwise comparison of DBF-scores based on different models. Each plot comprises DBF-scores of 27,628 *Arabidopsis thaliana* ORFs in a pairwise comparison of two different models.


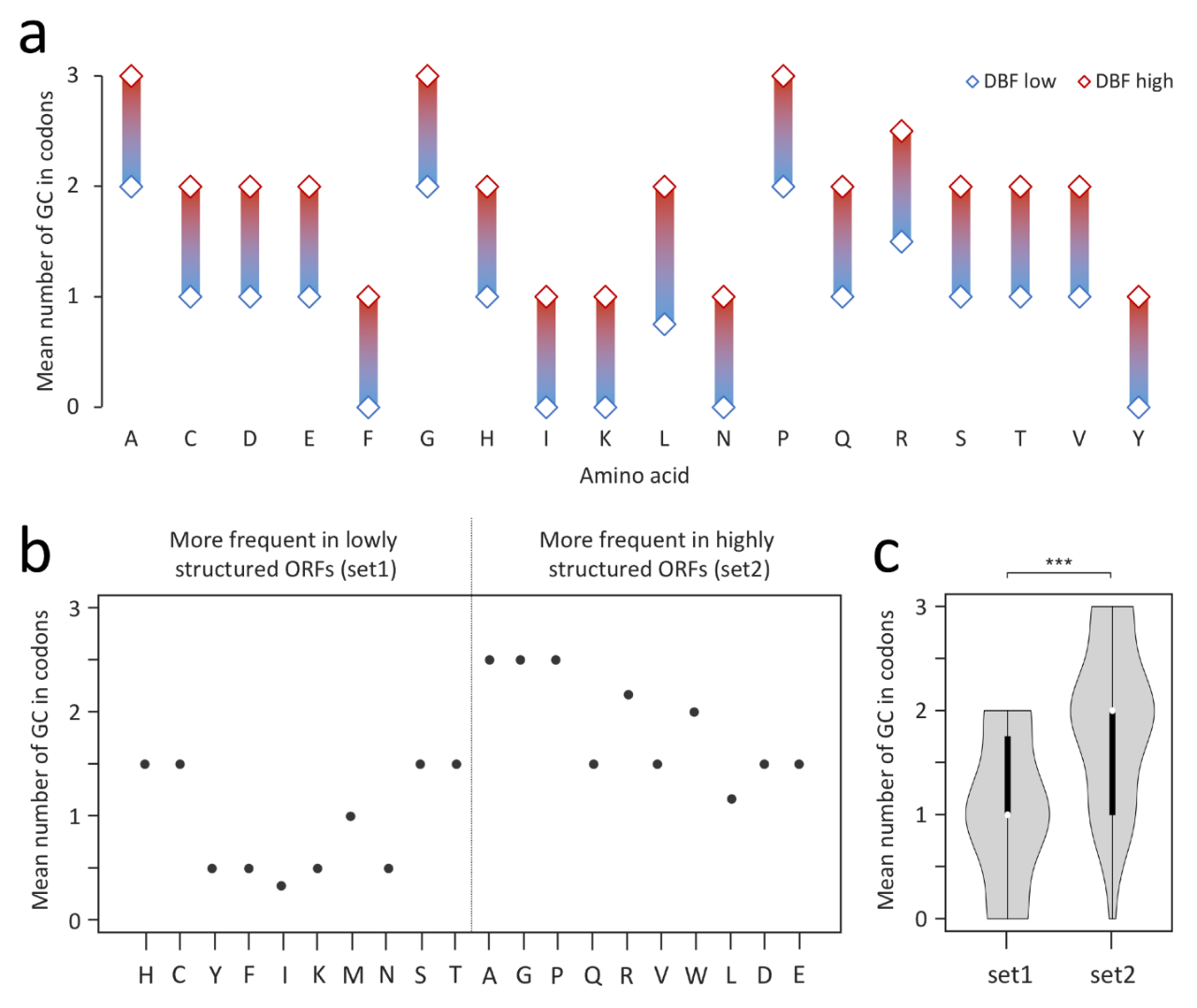


**Supplementary Figure 2.** Codon GC-content and ORF structuring. a) Mean GC content of codons for each amino acid divided into those that are found more frequently in highly structured oORFs (DBF high) and those that are found more frequently in lowly structured oORFs (DBF low) using exemplarily data from Mus musculus (see figure 3c). b) Mean GC content of codons for each amino acid sorted by DBF score (see figure 3d). c) Mean GC content of codons for amino acids of set 1 compared to set 2 (see figure 3d). ***: p<0.001 (Mann-Whitney-U test).
